# Usutu virus promotes West Nile virus replication in *Culex pipiens* biotype *molestus* during simultaneous and sequential co-infections

**DOI:** 10.1371/journal.ppat.1014601

**Published:** 2026-09-11

**Authors:** Christin Körsten, Cornelia Silaghi, Mandy Schäfer

**Affiliations:** Friedrich-Loeffler-Institut – Federal Research Institute for Animal Health (FLI), Institute of Infectology, Greifswald - Insel Riems, Germany; Washington State University, UNITED STATES OF AMERICA

## Abstract

The epidemiological co-circulation of the two flaviviruses West Nile virus (WNV) and Usutu virus (USUV) in several European countries poses the risk of co-infections in vertebrate hosts and mosquito vectors. WNV is an important human pathogen, whereas USUV is primarily relevant in veterinary and wildlife health. Co-infections have been detected in birds and, occasionally, in humans. To determine the potential consequences of co-exposure and co-infections in mosquitoes on viral transmission, lab-reared *Culex pipiens* biotype *molestus* were orally infected with both viruses either simultaneously or sequentially at 7-day intervals with German isolates of WNV lineage 2 and USUV lineage Europe 3. Simultaneous exposure resulted in a significantly increased WNV infection rate, while infection and transmission of USUV were inhibited at the same time. During sequential exposure, prior WNV exposure also had a negative effect on susceptibility to USUV, whereas conversely, WNV infection rates were not altered by prior USUV exposure. Furthermore, mosquitoes with established co-infections after simultaneous co-exposure exhibited higher WNV viral loads than those infected exclusively with WNV. The study reveals complex interactions between WNV, USUV, and the mosquito vector, which could influence vector competence and vector capacity of *Culex pipiens* biotype *molestus* in areas with co-circulation of WNV and USUV.

## Author summary

West Nile virus and Usutu virus are two closely related flaviviruses transmitted by mosquitoes. Both viruses share a very similar transmission cycle and often occur in the same regions. Consequently, co-infections with both viruses are possible in vertebrates as well as in mosquitoes. To investigate potential consequences of these co-infections in mosquitoes on transmission of both viruses, we infected mosquitoes of the species *Culex pipiens* biotype *molestus* with both viruses simultaneously or at a 7-day interval via virus-containing blood feeding. We were able to demonstrate that West Nile virus benefits from simultaneous exposure to Usutu virus, especially in the case of established co-infections. At the same time, a simultaneous or prior exposure to West Nile virus inhibits replication of Usutu virus. These interactions between both viruses and the mosquito can have a crucial impact on viral transmission.

## Introduction

The increasing geographic circulation of arthropod-borne viruses (arboviruses) poses new challenges to global public and veterinary health [1,2]. Beyond health risks associated with single arboviral infection, co-infections with multiple arboviruses can present diagnostic and therapeutic problems in affected areas [3,4]. Their consequences in vertebrate hosts are diverse, ranging from cross-protection to more severe diseases driven by higher viral load and antibody-dependent enhancement [3,5–9]. At the same time, co-infections in mosquito vectors can likewise influence transmission dynamics in multiple ways, as reviewed in [10]: enhancement or reduction of both viruses, competition of both viruses, or no interaction at all. Such outcomes can further depend on the timing of co-infection (simultaneous or sequential) [11–13], the genetic viral lineages [14], the mosquito species [15], and even specific mosquito populations [16]. Laboratory co-infection studies in mosquitoes offer the opportunity to investigate these diverse interactions and thus draw a more informed conclusion for future surveillance and control strategies in regions with arbovirus co-circulation.

West Nile virus (WNV, *Orthoflavivirus nilense*) and Usutu virus (USUV, *Orthoflavivirus usutuense*) are closely related mosquito-borne flaviviruses that co-circulate across many African and European countries [17,18]. While WNV is of major public health importance, USUV primarily affects avian populations and only rarely causes human disease. Besides their geographic co-circulation, both viruses also share a similar transmission cycle [19,20]. Both viruses are primarily transmitted by mosquitoes of the genus *Culex*, with *Cx. pipiens* biotype *pipiens* and *Cx. torrentium* being the most important vector species in Europe [20,21]. Birds are the primary and reservoir hosts, while humans and other mammals can become infected but are considered dead-end hosts and do not contribute to transmission [19]. WNV, in particular, can lead to severe illness in humans [22,23], whereas USUV has zoonotic potential, but most human infections with USUV are asymptomatic, although symptomatic and neuroinvasive cases in immunocompetent individuals have been reported [24,25].

Co-infections with WNV and USUV in vertebrate hosts have already been reported in several European countries. In Germany, where WNV lineage 2 and multiple lineages of USUV are co-circulating [26,27], cases of simultaneous infections have been detected in birds [28] and a confirmed WNV/USUV co-infection was identified in a human blood donor in 2020 by nucleic acid testing [29]. In other European countries, further confirmed co-infections have been documented in birds [30], and an additional human WNV/USUV co-infection was reported in an asymptomatic blood donor in Austria in 2018 [31]. In addition, serologic indications of exposure to both viruses have been reported in humans and horses, although this does not allow conclusion on the occurrence of co-infections [32–34]. In contrast, no co-infections have been detected in a single mosquito to date. Both WNV and USUV were simultaneously detected in several pools of *Cx. pipiens* mosquitoes collected in Slovakia, indicating co-circulation and suggesting the possibility of co-infected individuals [35]. However, the impact of such co-infections on the transmission dynamics of WNV and USUV in regions with co-circulation remains unclear.

Previous *in-vitro* co-infection studies have shown that WNV can suppress USUV replication in mammalian, avian and mosquito cells [11,36]. Simultaneous co-exposure in different mosquito species, however, indicated that viral interaction between WNV and USUV may be species-dependent. For instance, previous laboratory studies in mosquitoes using European strains of WNV lineage 2 and USUV Africa 3 showed reduced susceptibility to USUV in *Cx. pipiens* biotype *pipiens*, unaltered infection and transmission in *Cx. pipiens* biotype *molestus* and increased susceptibility to USUV in *Aedes vexans* [11,15]. In addition, sequential co-infection experiments in *Cx. pipiens* biotype *pipiens* showed that a prior USUV Africa 3 infection can reduce susceptibility to WNV lineage 2. However, only a prior exposure to USUV was tested in this study, while the reverse order was not assessed [11]. These studies were further limited to USUV Africa 3. In contrast, USUV lineage-specific effects on virus–virus interactions remain insufficiently explored, despite the co-circulation of different USUV lineages in Europe [26]**.** In order to reflect the dynamics in the European mosquito populations, additional co-infection studies with other USUV lineages are needed, as viral lineage may significantly influence interaction outcomes [14,37,38]. Therefore, in the present study, we used German strains of WNV lineage 2 and USUV Europe 3 to investigate both simultaneous and sequential co-exposures in *Cx. pipiens* biotype *molestus*, with the aim to assess the impact of USUV lineage and infection order on infection, dissemination, and transmission dynamics in mosquitoes.

## Results

### Virus exposure might affect mosquito fitness and feeding behavior

During the simultaneous co-exposure studies, control groups from the same mosquito population and generation were included and exposed to only one virus. No statistically significant differences were observed in these experiments regarding blood uptake or survival rates after single or double virus exposure (S1 Table).

For the sequential co-exposure studies, infectious blood was offered to the mosquitoes via cotton sticks during exposure I. A control group from the same population and generation was offered non-infectious blood at the same time. Seven days later, during exposure II, all groups were offered virus-spiked blood. Prior exposure to WNV had no effect on the blood-feeding behavior (43.06% and 44.44%; Chi-square test, *p* = 0.659, S3 Table). In contrast, significant differences in feeding rates were observed after prior exposure to USUV: virus-exposed mosquitoes exhibited increased feeding behavior compared with those that had previously received non-infectious blood (41.91% and 27.22%; Chi-square test, *p* = 0.002, S3 Table).

**Fig 1:**
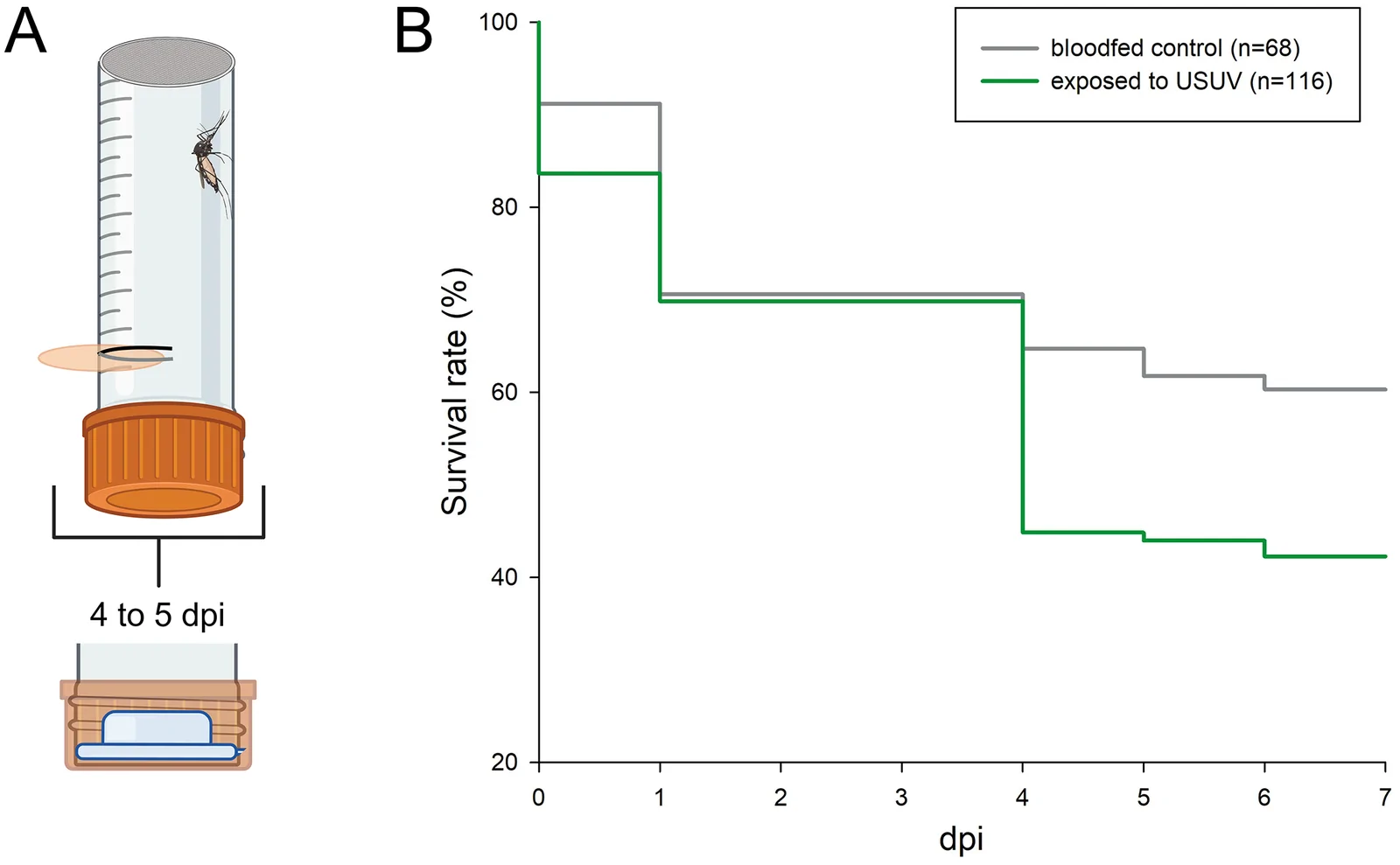
Increased mortality of mosquitoes exposed to USUV during oviposition period. **(A)** Schematic representation of the incubation method, including the possibility for egg laying. **(B)** Survival curve of mosquitoes from 0 to 7 days after infection (dpi) after USUV exposure, compared with a control group exposed to non-infectious blood. Created in BioRender. Körsten, **C.** (2026) https://BioRender.com/ttg99hh.

Initially, all engorged females were kept individually after exposure I, and were offered a container with water or moist cotton wool at 4 days post infection (dpi) for 24 hours to allow oviposition. However, a strikingly high mortality rate between 4–5 dpi was observed in the USUV-exposed group, resulting in a significantly reduced survival rate at 7 dpi (60.29% and 42.24%; Chi-square test, *p =* 0.027; Fig 1). Due to the high losses during oviposition, the incubation method was modified in subsequent experiments, which led to higher survival after exposure I (S4 Table).

After exposure II, co-exposed mosquitoes that first received WNV-spiked blood and, 7 days later, a blood meal containing USUV showed increased mortality compared with mosquitoes that did not feed on the USUV-spiked blood after WNV exposure (21 dpi survival rate of 31.94% and 51.11%; Chi-square test, *p* = 0.022). However, this effect was not observed after ingestion of WNV-spiked blood following prior USUV exposure compared to mosquitoes that did not feed again after USUV exposure (35.34% and 40.00%; Chi-square test, *p* = 0.440, S5 Table).

### Simultaneous exposure to WNV and USUV results in significant viral interactions characterised by USUV inhibition and enhanced WNV replication

To investigate viral interactions after simultaneous virus exposure, mosquitoes were offered blood containing USUV lineage Europe 3 and WNV lineage 2 in a 1:1 ratio and were analysed 14 days later. Mono-infections were carried out in parallel with mosquitoes from the same population and generation to allow comparison and to asses possible effects on the transmission of USUV and WNV. After single exposure, mosquitoes showed higher susceptibility to USUV than to WNV (infection rates of 95.83% and 70.83%; Fisher’s exact test, *p* = 0.048). However, in mosquitoes with established infection after single exposure, WNV loads in bodies, legs and wings were significantly higher than the respective USUV loads after single exposure (Mann-Whitney Rank Sum Test, *p* = 0.036; *p* = 0.010). No significant differences were observed in dissemination, transmission rates or transmission efficiencies (Table 1, S6 Table).

**Table 1:** Vector competence of *Cx. pipiens* biotype *molestus* for USUV and WNV after single and co-exposure. All experiments were performed at 26°C using a blood meal titer of 1.00 × 10^7^ TCID_50_/ml and a 14-day incubation period; sample sizes (n) are indicated.

Virus	Exposure	IR%(n/n)	DR%(n/n)	TR%(n/n)	TE%(n/n)
USUV	single	95.83(23/24)	73.91(17/23)	76.47(13/17)	54.17(13/24)
	co	37.50^a^(9/24)	55.56(5/9)	40.00(2/5)	8.33^a^(2/24)
WNV	single	70.83(17/24)	82.35(14/17)	57.14(8/14)	33.33(8/24)
	co	95.83^b^(23/24)	91.30(21/23)	66.67(14/21)	58.33(14/24)

IR, infection rate; DR, dissemination rate; TR, transmission rate; TE, transmission efficiency.

^a^ significantly lower than after single exposure (Fisher’s exact test, p ≤ 0.001).

^b^ significantly higher than after single exposure (Fisher’s exact test, p ≤ 0.05).

**Fig 2:**
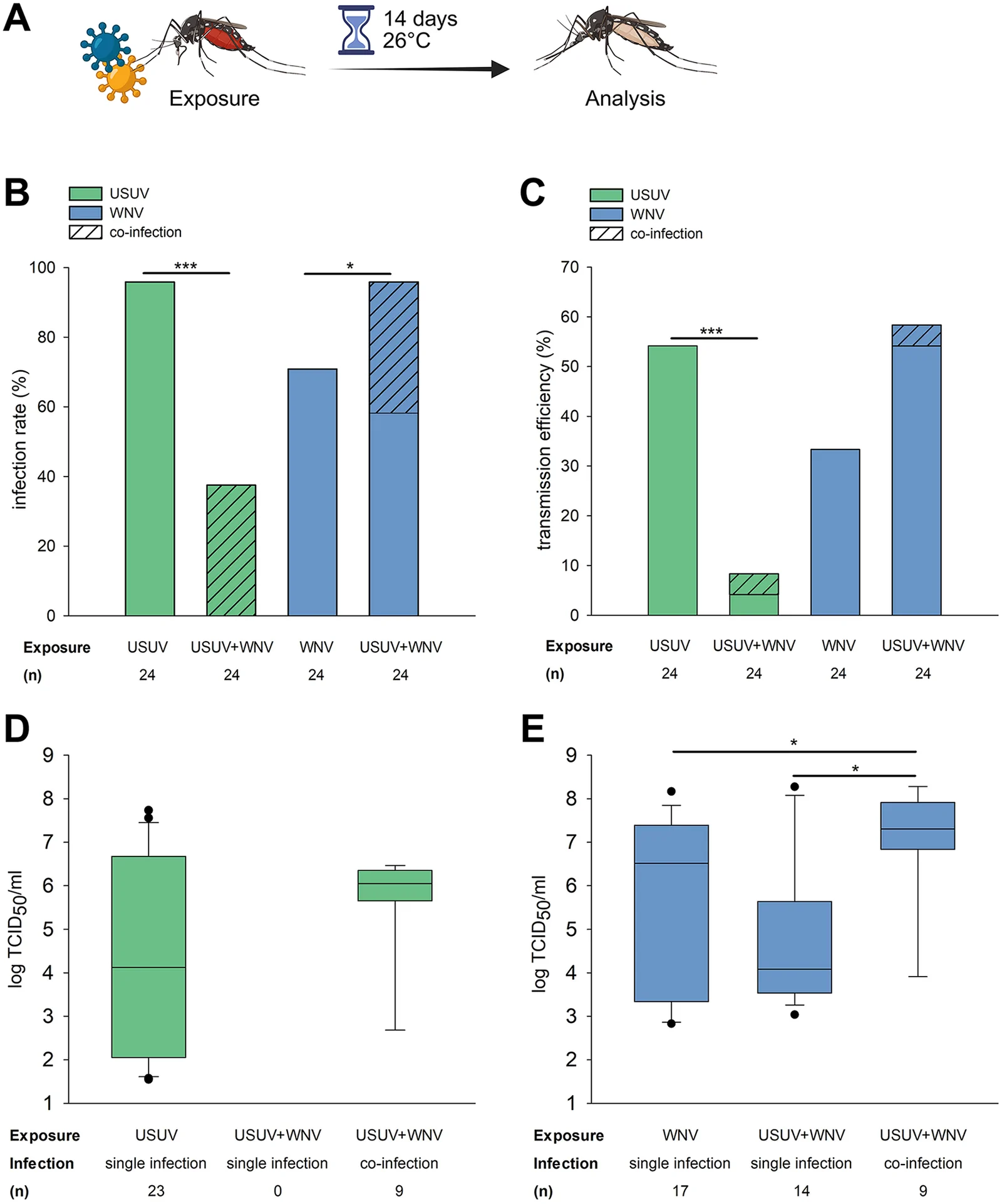
Viral interactions between USUV and WNV after co-exposure result in decreased USUV susceptibility and increased WNV replication. **(A)** Schematic representation of simultaneous co-infection studies. **(B)** Infection rates in mosquito bodies per surviving mosquitoes. **(C)** Transmission efficiencies in saliva containing viral RNA per surviving mosquitoes. **(D)** USUV body loads in mosquitoes after single and co-exposure. The x-axis indicates exposure type (USUV vs. USUV+WNV) and infection status at 14 dpi (USUV single infection vs. co-infection with WNV). **(E)** WNV body loads in mosquitoes after single and co-exposure. The x-axis indicates exposure type (WNV vs. USUV+WNV) and infection status at 14 dpi (WNV single infection vs. co-infection with USUV). (***) and (*) indicate statistically significance at p ≤ 0.001 and p ≤ 0.05, respectively. Created in BioRender. Körsten, **C.** (2026) https://BioRender.com/ttg99hh.

After simultaneous co-exposure to both viruses, mosquito susceptibility to USUV was significantly reduced compared with mosquitoes exposed only to USUV (Table 1, Fig 2B). In contrast, susceptibility to WNV was significantly higher following co-exposure with USUV (Table 1, Fig 2B). These effects also influenced the transmission of USUV, which was significantly reduced after co-exposure with WNV (Table 1, Fig 2C), whereas WNV transmission was not altered after single or co-exposure with USUV (Table 1, Fig 2C). Interestingly, USUV viral loads did not appear to be significantly altered after co-exposure to both viruses (Fig 2D). In contrast, co-infection resulted in significantly higher WNV viral loads in mosquito bodies compared with mosquitoes infected with WNV alone. (Fig 2E). Overall, an established co-infection was detected in 37.50% of mosquitoes that received both viruses. Notably, 100.00% of USUV-infected mosquitoes were simultaneously infected with WNV, whereas only 39.13% of all WNV-infected mosquitoes also exhibited USUV infection (Fig 2B). Exclusive transmission of USUV was observed in only one mosquito. Confirmed co-transmission, in which infectious particles of both viruses were detectable via cell culture, occurred in only one co-exposed mosquito (4.16%, Fig 2C).

### Prior exposure to WNV decreases susceptibility to USUV

To investigate potential effects on the transmission of WNV and USUV following two consecutive infectious blood meals, mosquitoes were offered blood containing one virus each, seven days apart, and were examined 14 days after the second exposure. As in the simultaneous co-infection studies, control groups were exposed to only one virus at the same time: at exposure I, mosquitoes receiving non-infectious blood were separated into blood-fed and non-fed groups and both were retained for further incubation. At exposure II, all remaining mosquitoes were offered infectious blood; individuals that successfully fed were classified according to their exposure I status as either co-exposed mosquitoes or control groups, while non-fed individuals at exposure II formed a second control group.

**Fig 3:**
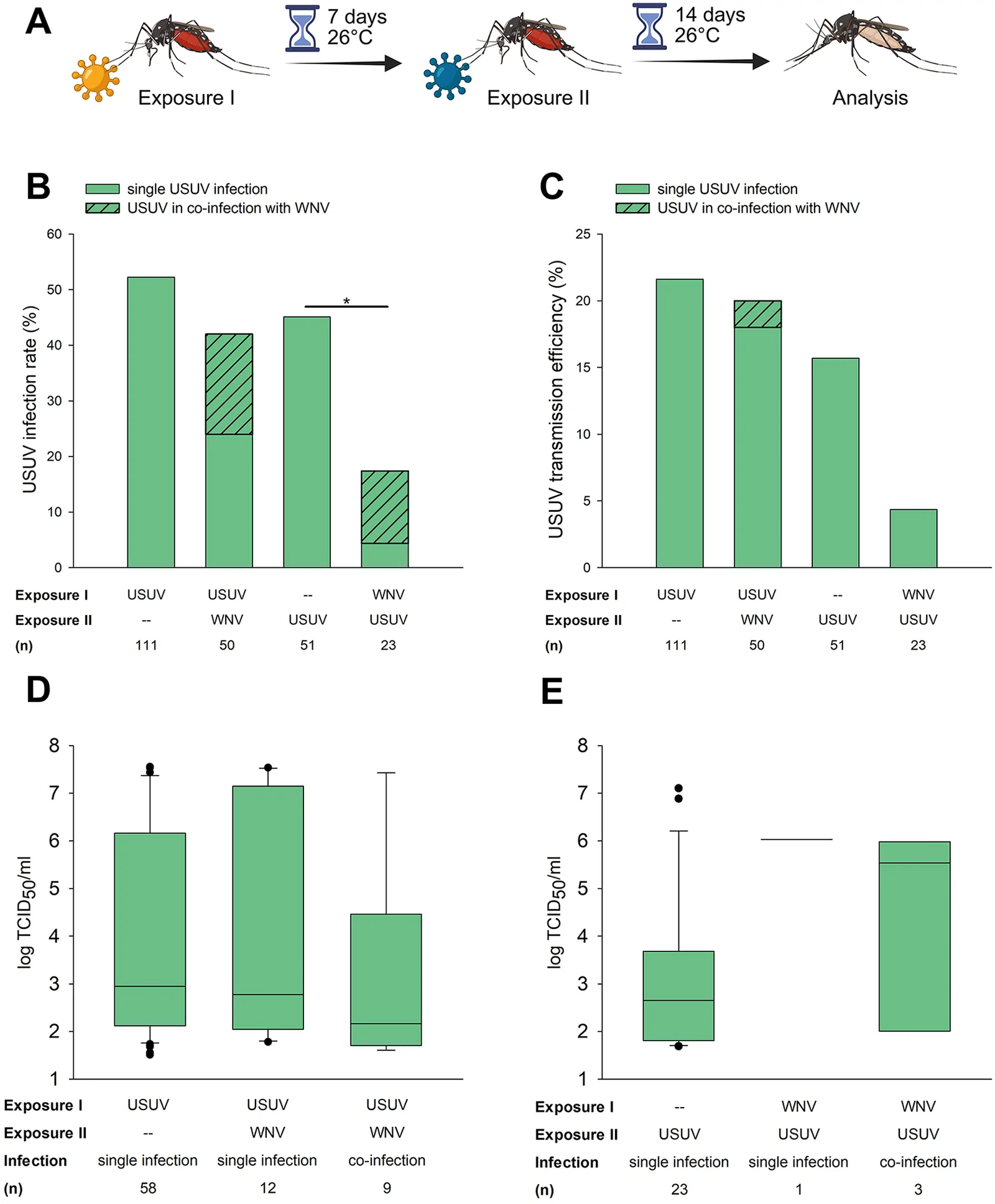
Sequential exposure to USUV and WNV results in decreased susceptibility to USUV after prior WNV-exposure. **(A)** Schematic representation of sequential co-infection studies. **(B)** USUV infection rates in mosquito bodies per surviving mosquitoes. **(C)** USUV transmission efficiencies in saliva containing USUV RNA per surviving mosquitoes. **(D)** USUV body loads in mosquitoes after single and sequential exposure. The x-axis indicates exposure (USUV with/without subsequent WNV exposure) and infection status at 21 dpi (USUV single infection vs. co-infection with WNV. **(E)** USUV body loads in mosquitoes with or without prior WNV exposure. The x-axis indicates exposure (USUV with/without prior WNV exposure) and infection status at 21 dpi (USUV single infection vs. co-infection with WNV). (*) indicate statistically significance with p ≤ 0.05. Created in BioRender. Körsten, **C.** (2026) https://BioRender.com/ttg99hh.

Mosquitoes that first received USUV-spiked blood showed no altered susceptibility or vector competence for USUV after subsequent WNV exposure compared with the control group (Table 2, Fig 3B and 3C). USUV body loads were also unaffected by subsequent ingestion of WNV, even in the cases of concurrently established WNV infection, which occurred in 42.85% of USUV-infected mosquitoes (Fig 3B and 3D). Interestingly, the dissemination rate of USUV was significantly reduced after subsequent WNV exposure (from 94.83% to 76.19%; Fisher’s exact test, *p* = 0.028; Table 2, S7 Table), although this reduction did not significantly affect transmission rates and transmission efficiency. Only one mosquito contained viral RNA from both viruses in its saliva, but infectious particles could be demonstrated only for WNV in this sample (Fig 3C, S7 Table, S8 Table).

**Table 2:** Vector competence of *Cx. pipiens* biotype *molestus* for USUV after single and sequential exposure to WNV. Mosquitoes were offered two blood meals 7 days apart and were analysed 14 days after second exposure (21 days in total). All experiments were performed at 26°C using blood meal titers of 1.00 × 10^7^ TCID_50_/ml; sample sizes (n) are indicated.

Exposure I	Exposure II	IR%(n/n)	DR%(n/n)	TR%(n/n)	TE%(n/n)
USUV	N/A	52.25(58/111)	94.83(55/58)	43.64(24/55)	21.62(24/111)
USUV	WNV	42.00(21/50)	76.19(16/21)	62.50(10/16)	20.00(10/50)
N/A	USUV	45.10(23/51)	60.87(14/23)	57.14(8/14)	15.69(8/51)
WNV	USUV	17.39(4/23)	100.00(4/4)	25.00(1/4)	4.35(1/23)

IR, infection rate; DR, dissemination rate; TR, transmission rate; TE, transmission efficiency; N/A, not applicable.

In experiments with reversed infection sequence, a prior non-infectious blood meal had no significant effects in USUV infection, transmission, or viral loads (S7 Table); therefore, all mosquitoes were combined into a single control group for comparison. Following prior WNV exposure, mosquito susceptibility to USUV was significantly reduced (from 45.10% to 17.39%; Fisher’s exact test, *p* = 0.036; Table 2, Fig 3B). Overall, after sequential co-exposure, only four mosquitoes were infected with USUV, three of which (75.00%) simultaneously harbored an established WNV infection. Transmission efficiency also appeared reduced after prior WNV ingestion, although not statistically significant (from 15.69% to 4.35%; Fisher’s exact test, *p* = 0.258; Table 2, Fig 3C). No evidence of potential co-transmission was detected during this sequence of virus exposure. USUV body loads showed no significant differences regardless of infection status or exposure history. However, the small sample sizes should be taken into account when interpreting these data (Fig 3E).

### Sequential co-exposure to USUV and WNV does not inhibit WNV and may promote WNV replication

**Fig 4:**
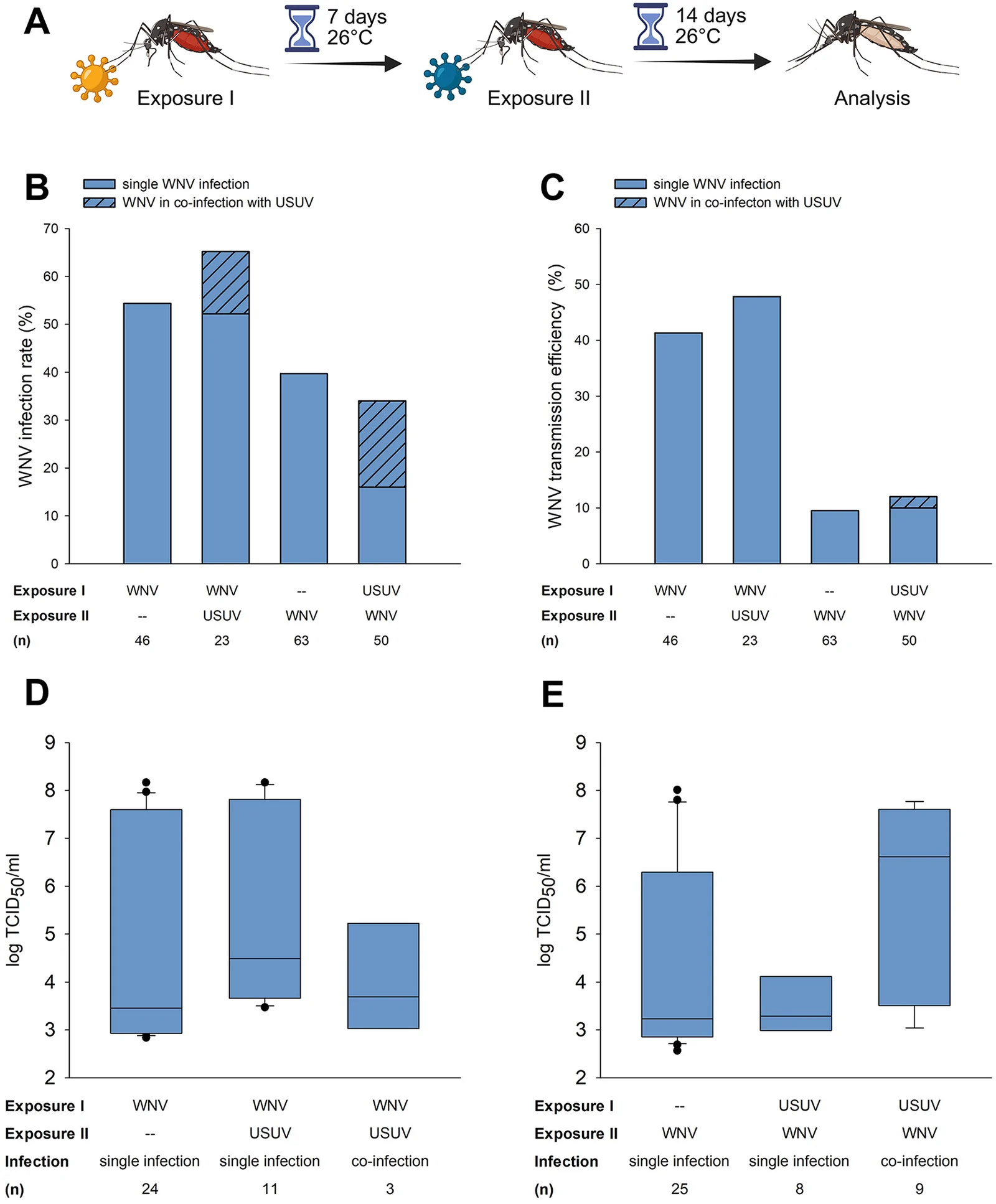
Sequential exposure to USUV and WNV does not affect WNV infection and transmission. **(A)** Schematic representation of sequential co-infection studies. **(B)** WNV infection rates in mosquito bodies per surviving mosquitoes. **(C)** WNV transmission efficiencies in saliva containing WNV RNA per surviving mosquitoes. **(D)** WNV body loads in mosquitoes with or without subsequent exposure to USUV. The x-axis indicates exposure (WNV with/without subsequent USUV exposure) and infection status at 21 dpi (WNV single infection vs. co-infection with USUV). **(E)** WNV body loads in mosquitoes with or without prior exposure to USUV. The x-axis indicates exposure (WNV with/without prior USUV exposure) and infection status at 21 dpi (WNV single infection vs. co-infection with USUV). Created in BioRender. Körsten, **C.** (2026) https://BioRender.com/ttg99hh.

Similar to the results for USUV vector competence, subsequent exposure to USUV had no measurable effect on WNV infection rates and viral body loads (Table 3, Fig 4B and 4D). Prior ingestion of a non-infectious blood meal also had no effect on WNV vector competence (S8 Table). Interestingly, prior exposure to USUV did not negatively affect WNV infection or transmission (Table 3, Fig 4B and 4C). Viral loads in mosquito bodies, analysed by infection status and exposure, showed no statistically significant differences (Fig 4E). However, when comparing WNV loads in bodies regardless of exposure, mosquitoes with established co-infection exhibited higher WNV load than those with single WNV infections with or without previous USUV exposure (Mann-Whitney Rank Sum Test, *p* = 0.027; S8 Table). Additionally, after co-exposure to both viruses, higher WNV loads in legs and wings were detected in mosquitoes with simultaneous disseminated USUV infection compared with mosquitoes with exclusive disseminated WNV infection (t-test, *p* = 0.033; S8 Table).

**Table 3:** Vector competence of *Cx. pipiens* biotype *molestus* for WNV after single and sequential exposure to USUV. Mosquitoes were offered two blood meals 7 days apart and were analysed 14 days after second exposure (21 days in total). All experiments were performed at 26°C using blood meal titers of 1.00 × 10^7^ TCID_50_/ml; sample sizes (n) are indicated.

Exposure I	Exposure II	IR%(n/n)	DR%(n/n)	TR%(n/n)	TE%(n/n)
WNV	N/A	54.35(25/46)	92.00(23/25)	82.61(19/23)	41.30(19/46)
WNV	USUV	65.22(15/23)	86.67(13/15)	84.62(11/13)	47.83(11/23)
N/A	WNV	39.68(25/63)	52.00(13/25)	46.15(6/13)	9.52(6/63)
USUV	WNV	34.00(17/50)	76.47(13/17)	46.15(6/13)	12.00(6/50)

IR, infection rate; DR, dissemination rate; TR, transmission rate; TE, transmission efficiency; N/A, not applicable.

## Discussion

Due to the co-circulation of USUV and WNV, there is a risk of co-exposure and co-infections in mosquito vectors, potentially affecting transmission dynamics. To address this issue, we investigated simultaneous and sequential co-exposure of *Cx. pipiens* biotype *molestus* with USUV Europe 3 and WNV lineage 2. Our results demonstrate that viral interactions depend on both viral lineage and sequence of virus exposure.

In previous experiments, mosquito larvae and adults from the same colony showed no reduction in fitness after exposure to a German USUV Africa 3 isolate and the same WNV lineage 2 isolate used in this study [15,39]. In contrast, the present study observed decreased survival shortly after USUV exposure. However, this effect was only observed when oviposition cups were provided, suggesting that the higher mortality occurred during the oviposition period. To our knowledge, this specific observation has not been previously reported. Notably, a related observation was made in the study by Styer et al., who demonstrated reduced fecundity in WNV-infected *Cx. tarsalis*, particularly during the first oviposition after exposure [40]. Although the mechanisms remain unknown, our findings suggest that processes associated with the first oviposition following infection may contribute to the reduced survival observed in USUV-exposed *Cx. pipiens* biotype *molestus*.

Together with the observed changes in survival during the oviposition period, altered blood-feeding behavior after viral exposure also suggests a potential impact on mosquito physiology. In this study, USUV-exposed mosquitoes exhibited an increased feeding rate during the second blood meal, similar to observations in WNV-infected *Cx. tarsalis* [40]*.* Likewise, Ciota et al. reported increased feeding rates in WNV-infected *Cx. pipiens* during the first blood meal 7 days after exposure, whereas this effect was not observed in later feedings [41]. The increased blood-feeding observed shortly after virus exposure may reflect physiological changes associated with an early infection, however, it remains unclear whether and to what extent the withdrawal of sugar prior to feedings influenced the mosquitoes’ feeding. Interestingly, the increased feeding was observed only after USUV exposure in our study, but not after WNV exposure. In addition, reduced survival was observed in mosquitoes exposed to USUV following prior WNV exposure, but not after reversed infection sequence, suggesting that USUV exposure had a greater impact on mosquito survival and feeding than WNV exposure. This difference may arise from the complex interaction between viral genetics, replication, mosquito metabolism and immune response [42,43]. Consistent with this, Ciota et al. demonstrated strain-dependent effects of WNV on mosquito survival, with reduced longevity observed only after exposure to a mosquito-adapted WNV strain, whereas the parental wild-type strain had no significant effect on survival [41]. These findings indicate that even small genetic differences between closely related viral strains can have distinct effects on mosquito physiology, suggesting that differences between USUV and WNV may also contribute to distinct mosquito responses. Beyond potential negative impacts of viral replication, mosquito resistance to USUV infection could also contribute to the observed exposure-associated effects, as reported by Ciota et al. and other studies [41,44,45]; however, as deceased mosquitoes were not examined, this remains speculative.

The differing effects of USUV and WNV uptake may also contribute to viral interactions during co-exposure and co-infection. Following simultaneous co-exposure, a significantly reduced USUV infection rate was observed, whereas WNV replication appeared to benefit. Based on the observed effects on mosquito fitness, USUV exposure may impose a greater burden on mosquitoes, potentially leading to altered metabolism, which in turn could positively influence WNV replication [42]. The inhibition of USUV, on the other hand, may result from direct viral interaction, for example, through competition for cellular components required for replication. After single exposure, higher viral loads of WNV were observed in infected mosquitoes, indicating that WNV can replicate more efficiently in *Cx. pipiens* biotype *molestus* and thus may have a competitive advantage over USUV. This is also supported by *in-vitro* experiments in which USUV was outcompeted by WNV in both vertebrate and mosquito cells, irrespective of viral lineage [11,36]. Interestingly, co-exposure of the same WNV isolate with the genetically distinct USUV lineage Africa 3 did not alter infection rates in the same mosquito colony [15], whereas the USUV Europe 3 strain used in this study produced a different outcome. This suggests a complex interaction between viral genotypes and the mosquito, which may vary among different viral lineages and strains [14]. It is also conceivable that co-exposure induced or enhanced antiviral immune pathways, such as the RNA interference (RNAi), JAK/STAT or Toll pathways [43], which may have been more effective against USUV than WNV. *In-vitro*, USUV Europe 3 showed slower viral growth compared with USUV Africa 3 in RNAi-competent *Cx. tarsalis* cells [36], suggesting that differential immune responses may affect viral replication and explain the observed variation. Additionally, flaviviruses possess multiple mechanism of immune suppression and evasion, such as the expression of sfRNA or the nonstructural protein 1 [46,47], which may act synergistically during co-exposure, primarily promoting WNV replication. This is further supported by the significantly higher WNV viral load observed in mosquitoes concurrently infected with USUV, potentially reflecting the combined influence of both viruses on the mosquito's defense response.

Unlike vertebrates, mosquitoes develop persistent infections following successful arbovirus infection, which, once established, are no longer cleared by the immune response [46,48]. In the case of sequential co-exposure, no effect on USUV or WNV was observed when the viruses were acquired during the first blood meal. Other studies have similarly shown that arboviruses are often unaffected by subsequent co-exposure [12,13,49,50]. These findings suggest that viral interactions within the mosquito may be most relevant during the early phase after exposure, before infections become established. On the other hand, several studies have reported effects of a prior infection on a subsequently acquired virus, often resulting in superinfection exclusion [11,13,49–51]. To date, superinfection exclusion between WNV and USUV has not been investigated in cell culture systems. However, in *Cx. pipiens* biotype *pipiens,* Wang et al. reported reduced WNV infection following USUV exposure, although the other exposure sequence was not examined [11]. In the present study, prior WNV exposure similarly affected subsequent USUV infection. Peng et al. demonstrated that impacts on the second virus may be due to altered metabolism and increased RNAi activity triggered by the first-acquired virus [49]. A similar mechanism may contribute to the altered USUV infection observed after sequential co-exposure, as after simultaneous co-exposure. Interestingly, in contrast to the study by Wang et al. [11], no negative effect on WNV was seen when the exposure order was reversed. In fact, there were even indications that an established USUV infection might have promoted WNV replication. Together, these findings highlight that superinfection exclusion during WNV-USUV co-exposure is not a universal outcome but may depend on multiple factors like virus genetics, exposure sequence and vector species.

Magalhães et al. observed positive effects of prior Chikungunya virus exposure on Zika virus transmission and discussed potential physical damage and an overwhelmed immune response as possible explanations [52]. Similarly, virus-induced changes in mosquito physiology could represent one possible explanation for the increased WNV loads observed after prior USUV exposure in our study. Although WNV has been shown to induce cellular degeneration and apoptosis in mosquito tissues [53–55], such effects were not assessed here. Therefore, whether USUV-induced physiological changes contribute to WNV replication remains speculative.

Overall, our results demonstrate that WNV-USUV interactions in *Cx. pipiens* biotype *molestus* are dependent on the virus lineage and exposure sequence. While prior or simultaneous USUV exposure did not impair WNV infection and was associated with increased WNV in co-infected mosquitoes after simultaneous exposure, USUV susceptibility was reduced after prior or simultaneous WNV exposure. However, it should be kept in mind that laboratory-reared mosquitoes were used in this study. Even though this offers the advantage of a homogeneous test group, these findings cannot be directly extrapolated to transmission dynamics in nature. Additionally, although *Cx. pipiens* biotype *molestus* are involved in the transmission of WNV and USUV, this biotype is not considered the main vector species. Nevertheless, these findings indicate that co-exposure to WNV and USUV does not result in a uniform outcome, but rather depends on multiple factors like virus genetics and infection dynamics. Given the co-circulation of multiple WNV and USUV lineages in Europe [26,56], and that several mosquito species are involved in transmission, further studies are needed to determine how these interactions influence arbovirus transmission under natural conditions.

## Materials and methods

### Mosquitoes, cells and viruses

The laboratory colony of *Cx. pipiens* biotype *molestus* originated from Hesse, Germany, in 2002 and has been maintained under laboratory conditions since. The rearing procedure was previously described [15]. Vero-B4 and Vero-76 cells, both derived from African green monkey (*Chlorocebus sabaeus*) kidney epithelial cells, were obtained from the Biobank of the Friedrich-Loeffler-Institut and maintained under standard cell culture conditions. The Vero-B4 cells were used for propagation of WNV, titration of the blood meals and for salivation assays. Since USUV could be propagated to higher titers in Vero-76 cells, this cell line was used for USUV propagation instead. The WNV lineage 2 strain “Germany 2018” (GenBank accession no. MH924836) was isolated from a Great grey owl (*Strix nebulosa*) in Germany in 2018 [57] and the 3^rd^ passage was propagated to a titer of 1.33 × 10^9^ TCID_50_/ml. The USUV Europe 3 strain “Germany 2011” (GenBank accession no. HE599647) was isolated from a Eurasian Blackbird (*Turdus merula*) in Germany in 2011 [58] and the 4^th^ passage was propagated to a titer of 5.62 × 10^7^ TCID_50_/ml.

### Simultaneous co-exposures

Simultaneous co-exposures were performed in two independent experiments as previously described [15]. Briefly, heparinised bovine blood obtained from cattle at the Friedrich-Loeffler-Institut was supplemented with 20µl of 5mM adenosine triphosphate (Merck, Darmstadt, Germany) per ml blood, and virus. For single-virus exposures, either 75µl 1:10 dilution of WNV or 178µl USUV stock were added per ml of blood to obtain a calculated titer of 1.00 × 10^7^ TCID_50_/ml; for co-exposures, both viruses were added. Subsequently, 7- to 14-day old, starved female mosquitoes were offered infectious blood meals provided via cotton-stick feeding. Blood-fed females were either individually sorted into small incubation chambers (28mm × 85mm; Carl Roth, Karlsruhe, Germany) or grouped in sets of up to 10 individuals in large incubation chambers (50mm × 100mm; Carl Roth), as modified in [15]. Two engorged females from each infection trial were collected immediately after blood feeding for subsequent RNA extraction and verification of infection. Mosquitoes were maintained for 14 days at 26°C, 85% relative humidity, and a 16:8 light-dark-cycle, corresponding to the standard conditions used for rearing this colony. These conditions were selected to minimise physiological stress caused by environmental changes and to allow efficient arbovirus replication, thereby enabling comparison with previous vector competence studies using similar conditions. Surviving females were dissected and forced to salivate as previous described [15,59]. In summary, legs and wings were removed and saliva was obtained by placing the proboscis into a filter tip containing 10µl PBS. One half of the obtained saliva sample was used for RT-qPCR, the other half was applied to Vero-B4 cell cultures to assess the presence of infectious viral particles, as described in [15]. Saliva was considered positive for infectious virus if a cytopathic effect was visible and viral RNA was detected in the supernatant of the cell culture. Results from individually incubated mosquitoes exposed to WNV or USUV in single exposure have already been published [60].

### Sequential co-exposures

For sequential co-exposures, a total of six (WNV exposure following USUV exposure) and four (USUV exposure following WNV exposure) independent experiments were conducted, respectively. For exposure I, 3- to 7-day old, starved females were offered either an infectious blood meal containing WNV or USUV, or a non-infectious blood meal via cotton-stick feeding. Due to initially low feeding rates in the non-exposed group, cell culture medium was subsequently added in the same proportion as the virus, resulting in comparably high feeding rates. Following feeding, non-engorged mosquitoes from the virus-exposed group were discarded. Mosquitoes from the non-exposed group were sorted into blood-fed and non-fed individuals. At 7 days post infection (dpi), all mosquitoes were offered an infectious blood meal containing either WNV or USUV.

**Fig 5:**
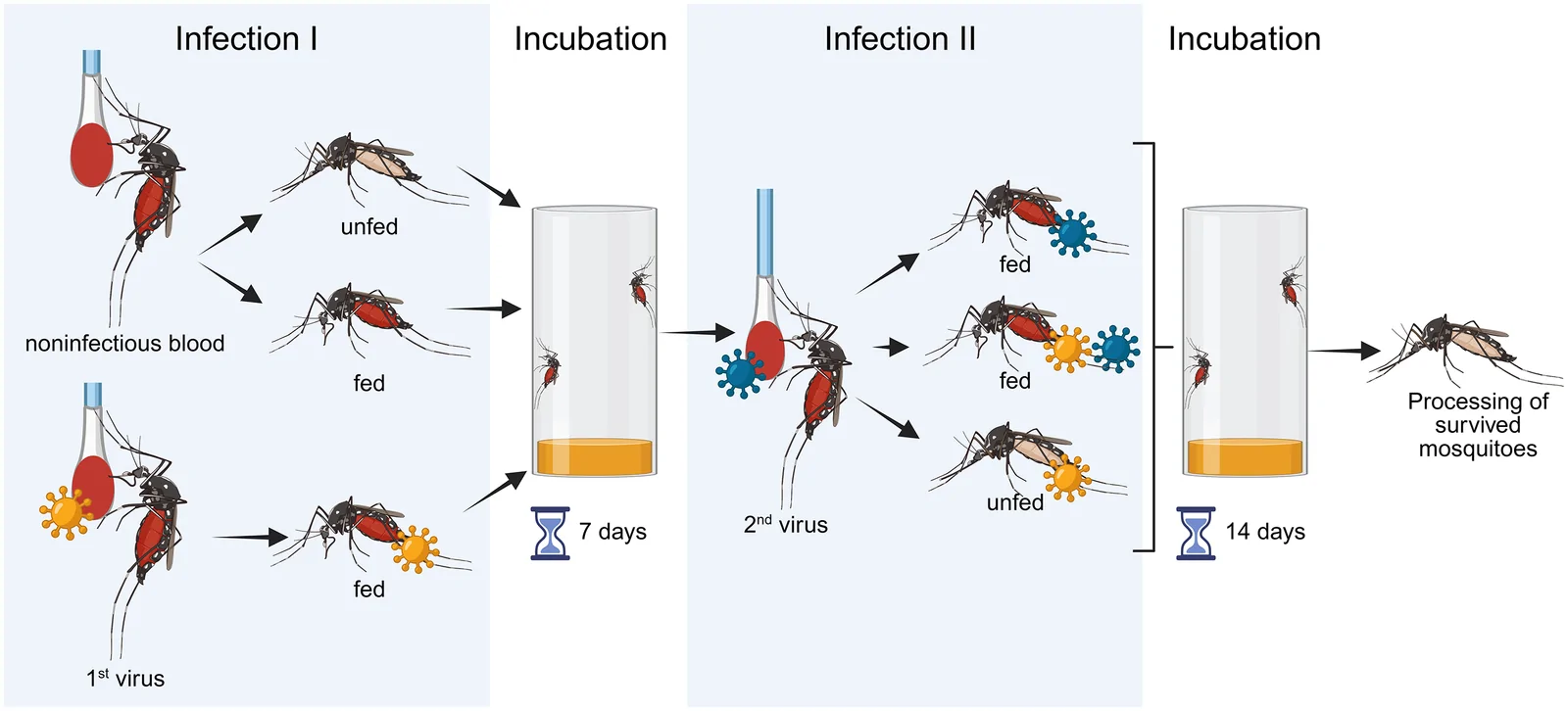
Workflow of sequential co-infections with USUV and WNV in *Cx. pipiens* biotype *molestus.* Created in BioRender. Körsten, **C.** (2026) https://BioRender.com/ttg99hh.

Initially, blood-fed mosquitoes from both groups were incubated individually in modified Falcon tubes [39] to provide an oviposition cup at 4 days post infection (dpi) for 24 hours (S1 Fig). For this purpose, lids of 5ml reaction vessels (Sarstedt, Nümbrecht, Germany) were filled with water or moist cotton wool and placed in the Falcon lids. The second blood meal was offered to these mosquitoes via a 50µl droplet [59]. However, this incubation method resulted in very high mortality rates, and the droplet feeding approach yielded very low feeding rates. Consequently, subsequent experiments were done as illustrated in Fig 5: Mosquitoes were incubated in groups of up to 10 individuals in large incubation chambers, and the second blood meal was offered to all groups via cotton stick feeding. After the second blood meal, mosquitoes were maintained for an additional 14 days. Incubation and processing of mosquitoes were performed as described for simultaneous co-exposures.

### Sample processing and molecular investigation

All samples (blood, whole mosquitoes, mosquito body parts, saliva, cell culture supernatant) for subsequent RNA extraction were collected into 560µl AVL buffer containing carrier RNA (Qiagen, Hilden, Germany). Whole mosquitoes, mosquito bodies and legs and wings were homogenised with a 3mm steel bead in a TissueLyser II (Qiagen) at 30 Hz for 2 minutes. Samples were stored at -80°C for at least 24h, then inactivated at 70°C for 10 minutes and centrifuged for 1 minute at 13000 rpm (Biofuge fresco; Heraeus instruments, Hanau, Germany). RNA was extracted from 200µl of the supernatant using the NucleoMag Vet Kit (Macherey-Nagel, Düren, Germany) according to the manufacturer’s instructions in a BioSprint 96 platform (Qiagen). Extraction efficiency was verified with an internal control [61]. Viral RNA was detected by RT-qPCR using the AgPath-ID Kit (Thermo Fisher Scientific, Massachusetts, USA) with primers and probe detecting the WNV 5’nontranslated region [62] and the USUV nonstructural protein 1 [63], as well as the internal control RNA [61]. Thermocycling conditions were as follows: 1 cycle at 48°C for 10 min, 95°C for 10 min, and 45 cycles at 95°C for 15 sec and 72°C for 30 sec. Based on results of a previous dilution series, samples with Cq values above 36 were considered negative [15].

### Vector competence indices

Feeding rates (FR) were calculated by dividing the number of blood-fed females by all living females that were offered blood. Survival rates (SR) were calculated by dividing all survived mosquitoes by all incubated mosquitoes. For sequential co-infections, the SR at 21 dpi refers only to mosquitoes that were alive and sorted after the second virus exposure. Infection rate (IR) is the percentage of infected mosquito bodies of all investigated mosquitoes. Dissemination rate (DR) refers to all mosquitoes where viral RNA was detected in legs and wings of all infected mosquito bodies. The transmission rate (TR) was calculated by dividing the number of mosquitoes with viral RNA and/or infectious virus in saliva samples by the number of mosquitoes with a disseminated infection. Transmission efficiencies (TE) were calculated by dividing the number of mosquitoes with viral RNA and/or infectious virus in saliva samples by the number of all investigated mosquitoes.

### Statistical analysis

Statistical analysis and graphics were generated with SigmaPlot 11 (Systat Software, Düsseldorf, Germany). Feeding and survival rates were compared using the Chi-square test, while Fisher’s exact test was used to analyse infection, dissemination and transmission rates as well as transmission efficiencies. Viral loads were compared using Student`s t-test or Mann–Whitney Rank Sum test, as appropriate. For comparisons involving more than two groups, a one-way ANOVA or the Kruskal–Wallis test was employed. Statistical significance was defined at a p value ≤0.05.

## Supporting information

S1 Fig Initial Workflow of sequential co-infections with USUV and WNV in *Cx. pipiens* biotype *molestus* with offering of oviposition cups 4 days after exposure I. Created in BioRender. Körsten, C. (2026) https://BioRender.com/ttg99hh.
(TIFF)

S1 Table Feeding and survival rates in *Culex pipiens* bioype *molestus* following single and simultaneous exposure. (XLSX)

S2 Table Feeding rates in *Culex pipiens* biotype *molestus* during exposure I. (XLSX)

S3 Table Feeding rates in *Culex pipiens* biotype *molestus* during exposure II. (XLSX)

S4 Table Survival rates in *Culex pipiens* biotype *molestus* after exposure I from 0 to 7 dpi. (XLSX)

S5 Table Survival rates in *Culex pipiens* biotype *molestus* after exposure II from 7 to 21 dpi. (XLSX)

S6 Table Vector competence and viral loads of *Culex pipiens* biotype *molestus* after single and simultaneous exposure to USUV and WNV. (XLSX)

S7 Table Vector competence and viral loads of *Culex pipiens* biotype *molestus* for USUV in single and sequential exposure with WNV. (XLSX)

S8 Table Vector competence and viral loads of *Culex pipiens* biotype *molestus* for WNV in single and sequential exposure with USUV. (XLSX)
