# Supplementary material for: Usutu virus promotes West Nile virus replication in Culex pipiens biotype molestus during simultaneous and sequential co-infections
Source: PLoS Pathog. 2026 Sep 11;22(9):e1014601. doi: 10.1371/journal.ppat.1014601 (PMC13607850; doi:10.1371/journal.ppat.1014601)
Supplement: Supplementary file 1 [file ppat.1014601.s011.docx]

**Response to Editor Comments**

Please address all reviewers' comments, especially those in relation to the relevance to human health.

Thank you for considering this manuscript. We have addressed all the reviewers' comments and revised the manuscript accordingly.

**Response to Reviewers**

**Response to Reviewer #1**

**Part I – Summary**

The authors present an interesting study about the influence of sequential and simultaneous co-infections of USUV and WNV in Cx. pipiens biotype molestus mosquitoes. Even though their findings are certainly interesting, the relevance of the results is insufficient for publication in PLoS Pathogens. The authors have already published a study on USUV and WNV co-infections (Körsten et al. 2023), in which they examined three species, including Cx. pipiens biotype molestus. In that study, they found no effect of co-infection on the vector competence in this species. The other two species examined showed effects, even if they showed opposite effects. It is unclear, and it is not discussed, why further experiments were conducted specifically with this species. Although the first study did not examine sequential co-infections, the results of this second study do not provide sufficient new insights to justify publication in PLoS Pathogens. Additionally, the relevance of co-infections with USUV and WNV is not well discussed. There are only very few reports of co-infections, it should be mentioned how often co-infections are reported in comparison to single infections. Importantly, USUV is not of relevance for human health, because the authors mistakenly give the impression USUV and WNV are relevant in the same way.

We thank the reviewer for the careful evaluation of our manuscript and for the constructive comments. We have addressed all points raised and revised the manuscript accordingly. We hope these revisions have strengthened the manuscript and addressed the reviewer's concerns.

**Part II – Major Issues: Key Experiments Required for Acceptance**

Line 20: don’t agree, USUV is not of major relevance to human health. USUV is also not particularly relevant to poultry farming, only wild birds are the ones major affected

We agree that WNV represents the greater public health concern and have revised the line to more clearly distinguish the public health relevance of WNV and USUV.

Line 30-32 + 45-47: delete this. Experiments are not detailed enough to confirm these hypotheses

Sentences were deleted accordingly.

Line 34: change “Culex pipiens” to “Culex pipiens biotype molestus”

Line was changed.

Line 48-49: it is not clear what is meant here, delete sentence

Line was deleted.

Line 51: delete “co-circulation” change to “circulation”, increasing circulation of arboviruses poses a risk itself, not the co-circulation. Citation 1+2 also don´t explain this wording.

This was changed.

Line 61: add “laboratory” co-infection studies

Added.

Line 64-65: rephrase, USUV is not of major relevance to human health. USUV is also not particularly relevant to poultry farming, only wild birds are the ones major affected

This was rephrased to distinguish the public health relevance of WNV and USUV

Line 68: Cx. pipiens biotype pipiens is considered as a main vector (not Cx pipiens biotype molestus), importantly: Cx. torrentium as an important vector is missing here

We agree that *Cx. pipiens* biotype *pipiens* and *Cx. torrentium* are considered the principal enzootic vectors of WNV and USUV in Europe. We have revised the Introduction accordingly and now explicitly acknowledge the role of *Cx. torrentium*.

Line 69: please rephrase! This sentence can lead to major misunderstanding > infection does not automatically mean that they serve as hosts. Humans don´t develop a high viraemia, they don´t serve as hosts.

Sentence was rephrased.

Line 69-70: citation is missing

Citation was added.

Line 70-72: rephrase. What do you want to say? Are there cases of USUV infection in humans without any serious pre-existing conditions that severely compromise the immune system? If yes, please mention here.

We have rephrased this sentence and included the documented symptomatic USUV infections in immunocompetent individuals.

Line 76: wrong citation. There is no report of an acute USUV-WNV-co-infection in this publication

We have corrected the wording to accurately reflect that the cited study reports detection of a WNV–USUV co-infection in a human blood donor, rather than a clinically described acute co-infection case.

Line 76-77: Rephrase! Make sure citations are correct cited (29-33). Don’t mix up serological results with co-infections!

We have revised the sentence to clearly distinguish between virological detections and serological evidence.

Line 78-80: rephrase, detection of viruses in wild caught mosquitoes do not prove an infection

The sentence was revised accordingly.

Line 85-92: please mention the origin of mosquito species and virus lineages (85-88), as you mention the importance of the investigation of European mosquitoes in line 89-92. Citation 15 is a study on European mosquitoes, indeed with the same USUV and WNV lineages that are mentioned here to be investigated. What is new in this manuscript in comparison to 15? Cx. pip. molestus is already investigated in 15. Focus on the new part of this study, it is not clear why do you performed this study by this sentence.

Thank you for this comment. We have revised this section to better highlight the novelty of our study. In contrast to our previous work, we used a different USUV lineage (Europe 3) and additionally investigated both simultaneous and sequential co-exposure scenarios.

Line 98: the whole part of “virus exposure alters mosquito fitness and feeding behavior” seems to undetailed to confirm any hypothesis. What is the total number of tested mosquitoes? How often are experiments repeated? Either delete this part or perform more experiments exactly the same way to confirm these hypotheses

We agree that the analysis of mosquito fitness and feeding behavior were not part of the primary study aims and should therefore be interpreted with caution. These observations emerged during the experiments and were consistently observed across independent replicates, and are supported by statistical analyses. We believe that reporting these findings is important, as they may provide relevant preliminary insights into virus-induced changes in mosquito behavior and fitness. However, we acknowledge that the study design and sample size were not specifically optimized for these kind of analysis. We have therefore revised the manuscript to clearly present these results as exploratory findings.

Line 106: explain “lower blood uptake”. Do you mean less volume or a lower feeding rate of all mosquitoes?

It is a lower feeding rate, we changed the wording to make that clear.

Line 103-116: rephrase this part, it is too confusing. It is not clear when each feeding rate was used, and in particular, it is not clear which ones are being compared. I would recommend to only show results of one method and delete the whole part of the other feeding method.

We deleted the comparison of different feeding methods to avoid further confusion and revised this section accordingly.

Line 142: add infection rates

Infection rates were added.

Line 142-144: rephrase

The sentence was rephrased for better understanding.

Line 149: add more informations (blood meal titer, incubation-period and -temperature, n)

Information was added as suggested.

Fig 2: what do you mean with “positivity rate”? Add incubation temperature

We agree that the term “positivity rate” may not be sufficiently specific without further context. In the revised figures, we have clarified the terminology and now explicitly indicate the respective rates (infection rate, dissemination rate, and transmission rate) in the figure axes and legends. We also added the incubation temperature.

Fig 2 D + E: It is not clear how the USUV+WNV single infection worked. Please explain in the legend or change graphic

We added an explanation in the figure legend.

178: Did the control group received the same amount of blood meals in the end?

Control mosquitoes were grouped based on their feeding status at each exposure rather than receiving identical blood intake across all groups. We have revised the section to more clearly describe this experimental design and the resulting control group structure.

Line 193-194: add more information, see table 1

Information was added as suggested

Fig 3: what do you mean with “positivity rate”? Add incubation temperature

Figure was revised as in Figure 2.

Line 222: please define “vector competence”. Transmission?

“Vector competence” was changed to “susceptibility and transmission” to avoid misunderstanding.

Line 231-232: add more information, see table 1

Information was added as suggested.

Fig 4: what do you mean with “positivity WNV rate”? Add incubation temperature

Figure was revised as in Figure 2.

Line 243-253: This is a summary of the complete result part. Delete and include in the discussion part in context to other studies, if not already concluded

We agree and have revised the opening part of the discussion accordingly. The revised paragraph now provides only a brief introduction to the main findings before discussing them in the context of previous studies.

Line 254-264: This assumption is not clear to the reader. Why would mosquitoes experience higher stress/mortality when an egg-laying sit is available than when there isn´t one? Delete or change.

We revised this section to explain this assumption better.

Line 265-278: The effect should be more discussed in context to other studies. For example: what kind of distinct effects did Ciota et al. observed?

We have expanded this section to discuss our findings in greater detail in the context of previous studies. In particular, we now describe the specific observations reported by Ciota et al., including the increase in blood-feeding and the strain-dependent effects on mosquito survival, and relate this findings to our observations.

Line 293: how much difference is in between USUV lineage 3 Europe/Africa? Who is more related USUV 2 Europe and USUV 3 Europe or both USUV 3?

Thank you for this comment. Our intention was not to imply a particular phylogenetic relationship between USUV Africa 3 and Europe 3 lineages based on their nomenclature. We have revised the text to clarify that the observed differences relate to the use of two genetically distinct USUV lineages, supporting the hypothesis that virus–virus interactions may be lineage-dependent.

Line 296: which mechanism do you mean, please go in more detail

We agree that this statement was too unspecific. We have revised the text to clarify that we refer to antiviral mosquito immune responses and now provide examples of relevant pathways.

Line 309-311: Tone down the statement a bit, it´s a guess, not a fact

We agree that the original statement was too strong. We have revised the sentence to indicate that the timing of virus–virus interactions during early infection is a possible explanation rather than a demonstrated mechanism.

Line 312-320: What about superinfection exclusion in cell culture experiments, are there any studies on WNV/USUV? Please discuss!

Thank you for this comment. We revised this part and clarified that, to our knowledge, sequential WNV/USUV infections have not been investigated in cell culture systems, but in mosquitoes by Wang et al., and we expanded the discussion in association with this study.

Line 324-326: Where is the point of this for this discussion? You didn´t observed apoptosis due to WNV infection at all.

We agree that the original wording was too speculative for a mechanism that was not investigated in our study. We have revised the section to clarify that tissue damage and apoptosis represent possible mechanisms described in other systems, but that their relevance for USUV–WNV interactions in mosquitoes remains unknown.

Line 324-330: delete this part or rephrase

This part was revised.

Line 331-339: The conclusion of this study is very vague and unclear for the reader. Rephrase.

Thank you for this comment. We agree that the previous conclusion was too general. We have revised this section to more clearly highlight the main findings of the study, and that the consequences for transmission may vary under different epidemiological scenarios.

344-345: please provide more details about the cell line

We added more details about Vero-B4 and Vero-76.

345: more details! Isolated from which animal/human, etc.

More details were added.

346: Is this the same cell line? Provide details

More details were added.

347: see 345

More details were added.

348: provide details about Vero-B4

More details were added.

342-349: Why did you used three different Vero cell lines? Explain in the text

The explanation why both Vero-B4 and Vero-76 were used instead of only one Vero cell line was added.

350: how often do you performed this experiment? Add information

Information was added.

353: origin of blood?

Information was added.

351-354: how much percentages of what?

Amount of added ATP and viruses were added.

Table S9: is not necessary to provide this data in the manuscript, delete

Thank you for this comment. We agree that these data do not provide additional information relevant to the main objectives of the study. We have therefore removed Table S9.

360: Why do you choose this temperature? Explain! As far as average temperatures in Germany are concerned, the selected temperature seems to be too high. Why 85% humidity? This is also a very high humidity value, what´s the reason?

We have clarified in the manuscript that the temperature and humidity were chosen to maintain the established rearing conditions of the mosquito colony. While these conditions may not reflect average environmental conditions in Germany, they provide standardized conditions that support efficient virus replication and allow comparison with previous studies.

361: delete information about incubator, not necessary

Deleted.

368: how often do you performed this experiment? Add information

Information was added.

Fig 5: not necessary, as the workflow is also shown in Fig 4. Either delete Fig 4A or Fig 5.

Thank you for this comment. We agree that both figures show related experimental workflows. However, Fig. 4A serves as a simplified graphical abstract of the experimental design to facilitate interpretation of the corresponding results, whereas Fig. 5 provides a detailed methodological workflow, including the timing and sequence of experimental steps. Since the Results section precedes the Materials and Methods section, we consider both figures useful for different purposes and have kept both.

392: there are too much details in this part, but important ones are missing: which qPCR-Kit/protocol did you use. It is not enough to cite a paper where it is mentioned.

We added the information on PCR-Kit and the protocol.

402-403: Please mention the reason, citing is not enough

The reason for the cut-off was added.

405 and 408-410: the sentences have the same meaning, delete one.

This part was revised.

405-410: Add information how to calculate rates!

Information on calculation was added.

**Part III – Minor Issues: Editorial and Data Presentation Modifications** (No Response)

**Response to Reviewer #2**

**Part I – Summary**

This paper delves into a complicate and relatively understudied part of vector competence. While it is expanding on previous works with related virus lineages, as seen in the results, one WNV or USUV lineage cannot be used to describe all others in these complex interactions. This work is important for our understanding of the transmission of these viruses but is difficult to do, which should be considered when examining the few weaknesses of the study.

We thank the reviewer for the evaluation of our manuscript and for the constructive comments. We have addressed all comments and revised the manuscript accordingly.

**Part II – Major Issues: Key Experiments Required for Acceptance** (No Response)

**Part III – Minor Issues: Editorial and Data Presentation Modifications**

Results: In table 1, it would be helpful to the reader to include a for the two Infection Rates.

Thank you for this suggestion, we added the denotation of significance.

Discussion: Line 332 - Results do not necessarily indicate outcompeting only that there is inhibition of WNV infection. Please include in the discussion the two following caveats - Well established mosquito colonies differ from field mosquito populations is a number of ways (genetics and gut microbiome) that may impact how the trends and results in this study translate. Sugar staving mosquitoes can impact blood feeding behavior that may complicate the results described in the study.

We revised the discussion accordingly to include your suggestions.

Methods: Please briefly describe how you determined which of the two viruses was infectious in the presence of both WNV and USUV RNA (Ln 186). It can be found in the key at the bottom of S7 and S8 tables, but should be made clear in the manuscript.

Thank you for the suggestion, the information was added accordingly.

**Response to Reviewer #3**

**Part I – Summary**

The manuscript describes the results of a series of experiments that investigated how two related flaviviruses (USUV and WNV) may compete inside the mosquito vector when they enter the mosquito body either simultaneously or sequentially. The experimental design was solid (including controls and well explained experimental procedures), and data are presented in a clear and understandable way. However, studies on the impact of co-infections on mosquito fitness and vector competence are not new and have been widely studies at different levels. The novelty of the present study lies in the fact that the authors used a different lineage for USUV in combination with a WNV strain that is circulating in Europe. I have a few remarks to further improve the manuscript.

We thank the reviewer for the evaluation of our manuscript and for the constructive comments. We have addressed all comments and revised the manuscript accordingly.

**Part II – Major Issues: Key Experiments Required for Acceptance** (No Response)

**Part III – Minor Issues: Editorial and Data Presentation Modifications**

1. Title: leave out ‘viral wingmen’. In my opinion, it has no logical link with the topic of the study. Without this short ‘popular’ part, the title covers the content of the paper nicely.

Thank you for this suggestion, we removed this part of the title.

2. Abstract, line 20: replace ‘medicine’ with ‘health’.

This was replaced.

3. The abstract mentions that ‘…mosquitoes with established co-infections after simultaneous and sequential co-exposure exhibited higher WNV viral loads.’. While this is indeed apparent for simultaneous exposure (Figure 2E), I do not see this indicated for the sequential exposure data (Figure 4E). However, the text in lines 226-227 mention that ‘mosquitoes with established co-infection exhibited higher WNV load than those with single WNV infections, p = 0.027). How is this difference visible in Figure 4E? I had some trouble in understanding what was compared with what.

Thank you for pointing this out. We agree that the statement in the abstract was too general and could be misleading, as the increase in WNV viral loads was not consistently observed when comparing each sequential exposure group individually. We have therefore removed this statement from the abstract and revised the wording to better reflect the exposure-dependent effects observed in our study.

4. Line 30: ‘then’ should read ‘than’

This was changed

5. Line 64/65: remove ‘the’ before the virus names

Removed.

6. Results section: in many instances, just the p-values are reported without providing further details on the outcomes of the actual statistics. These details should be included throughout the results.

The details were added.

7. L.156: if it is not significantly different, you cannot claim that ‘WNV was transmitted more frequently’. This could be co-incidence as confirmed by your statistical test.

We agree that this statement was not sufficiently supported by our results, and we have revised this part accordingly.

8. Figure 3: make the difference between panels B and C clear in the Y-axis labels. Both now mention USUV positivity rate, but B is for bodies and C is for saliva.

This was changed.

9. L. 210: here the authors mention that susceptibility to USUV was significantly reduced, but without providing any further statistical details on the outcome of the test (and the type of test performed).

We have revised this section to provide additional information on the statistical analysis, including the statistical test performed and the corresponding outcome.

10. L. 341: mention passage number of the viral strains used here.

Added.

11. L. 341: why did the authors chose to use the molestus biotype? In literature, it is not really considered an enzootic vector of USUV or WNV and there is evidence that its vector competence is different (lower) for these viruses in comparison with the pipiens biotype. A critical discussion on this is missing.

Thank you for this comment. We have expanded the Discussion to better justify the use of *Cx. pipiens* biotype *molestus*. Although this biotype is generally considered less important than *Cx. pipiens* biotype *pipiens* and *Cx. torrentium* for the enzootic transmission of WNV and USUV, it is a competent vector under laboratory conditions and represents an established experimental model.

12. L 343: add ‘The’ before ‘rearing procedure’

Added.
