# Supplementary material for: Usutu virus promotes West Nile virus replication in Culex pipiens biotype molestus during simultaneous and sequential co-infections
Source: PLoS Pathog. 2026 Sep 11;22(9):e1014601. doi: 10.1371/journal.ppat.1014601 (PMC13607850; doi:10.1371/journal.ppat.1014601)
Supplement: Supplementary file 2 [file ppat.1014601.s012.docx]

**Response to Reviewers**

Dear Editor and Reviewers,

We sincerely thank the Editor and the Reviewers for their careful evaluation of our manuscript and for their constructive comments. We have revised the manuscript accordingly. Below, we respond to each comment and indicate the changes made.

**Reviewer #2**

**Comment 1 (Lines 73–77):** In the section, as written there appears to be two human blood donors with co-infections of WNV and USUV. However, when digging into the citation, both lead to the same case. Please rewrite to clarify the number of recorded human co-infections.

**Response:** Thank you for pointing this out. We re-examined the relevant literature and have revised the wording in the manuscript to clarify the number of documented human co-infections. To date, two asymptomatic human co-infections with West Nile virus (WNV) and Usutu virus (USUV) have been documented among blood donors: one in an Austrian blood donor in 2018 (31) and one in a German blood donor in 2020 (29). In both cases, co-infection was confirmed by nucleic acid testing.

**Comment 2 (Lines 79–82):** These sentences deliver the same information. They should be combined to increase reader clarity.

**Response:** Thank you very much for the suggestion. We have retained the distinction between the two statements, as they refer to different types of evidence. Specifically, the term ‘co-infection’ refers to the simultaneous detection of WNV and USUV using nucleic acid tests, whilst the term ‘serologic indications of exposure’ refers to the detection of exposure to both viruses based on serological neutralising antibody tests.

**Reviewer #3**

No specific comments from Reviewer #3 were included in the material provided to us. Therefore, no corresponding revisions can be addressed here.

We appreciate the Reviewers’ helpful comments, which have improved the accuracy, clarity, and readability of the manuscript. We hope that the revisions satisfactorily address the concerns raised.

Sincerely,
Mandy Schäfer
