# Supplementary material for: Usutu virus promotes West Nile virus replication in Culex pipiens biotype molestus during simultaneous and sequential co-infections
Source: PLoS Pathog. 2026 Sep 11;22(9):e1014601. doi: 10.1371/journal.ppat.1014601 (PMC13607850; doi:10.1371/journal.ppat.1014601)
Supplement: Peer review history

# Peer review: Decision Letter 0

**DOI:** 10.1371/journal.ppat.1014601.r001

**Authors:** Matthias Schnell, Alan Goodman

30 Jun 2026

PPATHOGENS-D-26-00495

Viral wingmen: Usutu virus promotes West Nile virus replication in Culex pipiens biotype molestus during simultaneous and sequential co-infections

PLOS Pathogens

Dear Dr. Schäfer,

Thank you for submitting your manuscript to PLOS Pathogens. After careful consideration, we feel that it has merit but does not fully meet PLOS Pathogens's publication criteria as it currently stands. Therefore, we invite you to submit a revised version of the manuscript that addresses the points raised during the review process.

Please submit your revised manuscript by Aug 29 2026 11:59PM. If you will need more time than this to complete your revisions, please reply to this message or contact the journal office at plospathogens@plos.org. When you're ready to submit your revision, log on to https://www.editorialmanager.com/ppathogens/ and select the 'Submissions Needing Revision' folder to locate your manuscript file.

Please include the following items when submitting your revised manuscript:

* A letter that responds to each point raised by the editor and reviewer(s). You should upload this letter as a separate file labeled 'Response to Reviewers'. This file does not need to include responses to any formatting updates and technical items listed in the 'Journal Requirements' section below.

* A marked-up copy of your manuscript that highlights changes made to the original version. You should upload this as a separate file labeled 'Revised Manuscript with Track Changes'.

* An unmarked version of your revised paper without tracked changes. You should upload this as a separate file labeled 'Manuscript'.

If you would like to make changes to your financial disclosure, competing interests statement, or data availability statement, please make these updates within the submission form at the time of resubmission. Guidelines for resubmitting your figure files are available below the reviewer comments at the end of this letter. As the corresponding author, your ORCID iD is verified in the submission system and will appear in the published article. PLOS supports the use of ORCID, and we encourage all coauthors to register for an ORCID iD and use it as well. Please encourage your coauthors to verify their ORCID iD within the submission system before final acceptance, as unverified ORCID iDs will not appear in the published article. Only the individual author can complete the verification step; PLOS staff cannot verify ORCID iDs on behalf of authors.

We look forward to receiving your revised manuscript.

Kind regards,

Alan G. Goodman

Academic Editor

PLOS Pathogens

Matthias Schnell

Section Editor

PLOS PathogensSumita Bhaduri-McIntosh

Editor-in-Chief

PLOS Pathogens

orcid.org/0000-0003-2946-9497

Michael Malim

Editor-in-Chief

PLOS Pathogens

orcid.org/0000-0002-7699-2064

**Additional Editor Comments (if provided):**

Please address all reviewers' comments, especially those in relation to the relevance to human health.

**Journal Requirements:**

If the reviewer comments include a recommendation to cite specific previously published works, please review and evaluate these publications to determine whether they are relevant and should be cited. There is no requirement to cite these works unless the editor has indicated otherwise.

1) Please amend your detailed Financial Disclosure statement. This is published with the article. It must therefore be completed in full sentences and contain the exact wording you wish to be published.

i) State the initials, alongside each funding source, of each author to receive each grant. For example: "This work was supported by the National Institutes of Health (####### to AM; ###### to CJ) and the National Science Foundation (###### to AM)."

ii) State what role the funders took in the study. If the funders had no role in your study, please state: "The funders had no role in study design, data collection and analysis, decision to publish, or preparation of the manuscript."

iii) If any authors received a salary from any of your funders, please state which authors and which funders..

If you did not receive any funding for this study, please simply state: u201cThe authors received no specific funding for this work.u201d

2) We have amended your Competing Interest statement to comply with journal style. We kindly ask that you double check the statement and let us know if anything is incorrect.

**Reviewers' Comments:**

Reviewer's Responses to Questions

**Part I - Summary**

Please use this section to discuss strengths/weaknesses of study, novelty/significance, general execution and scholarship.

Reviewer #1: The authors present an interesting study about the influence of sequential and simultaneous co-infections of USUV and WNV in Cx. pipiens biotype molestus mosquitoes. Even though their findings are certainly interesting, the relevance of the results is insufficient for publication in PLoS Pathogens.

The authors have already published a study on USUV and WNV co-infections (Körsten et al. 2023), in which they examined three species, including Cx. pipiens biotype molestus. In that study, they found no effect of co-infection on the vector competence in this species. The other two species examined showed effects, even if they showed opposite effects. It is unclear, and it is not discussed, why further experiments were conducted specifically with this species. Although the first study did not examine sequential co-infections, the results of this second study do not provide sufficient new insights to justify publication in PLoS Pathogens.

Additionally, the relevance of co-infections with USUV and WNV is not well discussed. There are only very few reports of co-infections, it should be mentioned how often co-infections are reported in comparison to single infections. Importantly, USUV is not of relevance for human health, because the authors mistakenly give the impression USUV and WNV are relevant in the same way.

Reviewer #2: This paper delves into a complicate and relatively understudied part of vector competence. While it is expanding on previous works with related virus lineages, as seen in the results, one WNV or USUV lineage cannot be used to describe all others in these complex interactions. This work is important for our understanding of the transmission of these viruses but is difficult to do, which should be considered when examining the few weaknesses of the study.

Reviewer #3: The manuscript describes the results of a series of experiments that investigated how two related flaviviruses (USUV and WNV) may compete inside the mosquito vector when they enter the mosquito body either simultaneously or sequentially. The experimental design was solid (including controls and well explained experimental procedures), and data are presented in a clear and understandable way. However, studies on the impact of co-infections on mosquito fitness and vector competence are not new and have been widely studies at different levels. The novelty of the present study lies in the fact that the authors used a different lineage for USUV in combination with a WNV strain that is circulating in Europe. I have a few remarks to further improve the manuscript.

**********

**Part II – Major Issues: Key Experiments Required for Acceptance**

Please use this section to detail the key new experiments or modifications of existing experiments that should be absolutely required to validate study conclusions.

Generally, there should be no more than 3 such required experiments or major modifications for a "Major Revision" recommendation. If more than 3 experiments are necessary to validate the study conclusions, then you are encouraged to recommend "Reject".

Reviewer #1: Line 20: don’t agree, USUV is not of major relevance to human health. USUV is also not particularly relevant to poultry farming, only wild birds are the ones major affected

Line 30-32 + 45-47: delete this. Experiments are not detailed enough to confirm these hypotheses

Line 34: change “Culex pipiens” to “Culex pipiens biotype molestus”

Line 48-49: it is not clear what is meant here, delete sentence

Line 51: delete “co-circulation” change to “circulation”, increasing circulation of arboviruses poses a risk itself, not the co-circulation. Citation 1+2 also don´t explain this wording.

Line 61: add “laboratory” co-infection studies

Line 64-65: rephrase, USUV is not of major relevance to human health. USUV is also not particularly relevant to poultry farming, only wild birds are the ones major affected

Line 68: Cx. pipiens biotype pipiens is considered as a main vector (not Cx pipiens biotype molestus), importantly: Cx. torrentium as an important vector is missing here

Line 69: please rephrase! This sentence can lead to major misunderstanding > infection does not automatically mean that they serve as hosts. Humans don´t develop a high viraemia, they don´t serve as hosts.

Line 69-70: citation is missing

Line 70-72: rephrase. What do you want to say? Are there cases of USUV infection in humans without any serious pre-existing conditions that severely compromise the immune system? If yes, please mention here.

Line 76: wrong citation. There is no report of an acute USUV-WNV-co-infection in this publication

Line 76-77: Rephrase! Make sure citations are correct cited (29-33). Don’t mix up serological results with co-infections!

Line 78-80: rephrase, detection of viruses in wild caught mosquitoes do not prove an infection

Line 85-92: please mention the origin of mosquito species and virus lineages (85-88), as you mention the importance of the investigation of European mosquitoes in line 89-92. Citation 15 is a study on European mosquitoes, indeed with the same USUV and WNV lineages that are mentioned here to be investigated. What is new in this manuscript in comparison to 15? Cx. pip. molestus is already investigated in 15. Focus on the new part of this study, it is not clear why do you performed this study by this sentence.

Line 98: the whole part of “virus exposure alters mosquito fitness and feeding behavior” seems to undetailed to confirm any hypothesis. What is the total number of tested mosquitoes? How often are experiments repeated? Either delete this part or perform more experiments exactly the same way to confirm these hypotheses

Line 106: explain “lower blood uptake”. Do you mean less volume or a lower feeding rate of all mosquitoes?

Line 103-116: rephrase this part, it is too confusing. It is not clear when each feeding rate was used, and in particular, it is not clear which ones are being compared. I would recommend to only show results of one method and delete the whole part of the other feeding method.

Line 142: add infection rates

Line 142-144: rephrase

Line 149: add more informations (blood meal titer, incubation-period and -temperature, n)

Fig 2: what do you mean with “positivity rate”? Add incubation temperature

Fig 2 D + E: It is not clear how the USUV+WNV single infection worked. Please explain in the legend or change graphic

178: Did the control group received the same amount of blood meals in the end?

Line 193-194: add more information, see table 1

Fig 3: what do you mean with “positivity rate”? Add incubation temperature

Line 222: please define “vector competence”. Transmission?

Line 231-232: add more information, see table 1

Fig 4: what do you mean with “positivity WNV rate”? Add incubation temperature

Line 243-253: This is a summary of the complete result part. Delete and include in the discussion part in context to other studies, if not already concluded

Line 254-264: This assumption is not clear to the reader. Why would mosquitoes experience higher stress/mortality when an egg-laying sit is available than when there isn´t one? Delete or change.

Line 265-278: The effect should be more discussed in context to other studies. For example: what kind of distinct effects did Ciota et al. observed?

Line 293: how much difference is in between USUV lineage 3 Europe/Africa? Who is more related USUV 2 Europe and USUV 3 Europe or both USUV 3?

Line 296: which mechanism do you mean, please go in more detail

Line 309-311: Tone down the statement a bit, it´s a guess, not a fact

Line 312-320: What about superinfection exclusion in cell culture experiments, are there any studies on WNV/USUV? Please discuss!

Line 324-326: Where is the point of this for this discussion? You didn´t observed apoptosis due to WNV infection at all.

Line 324-330: delete this part or rephrase

Line 331-339: The conclusion of this study is very vague and unclear for the reader. Rephrase.

344-345: please provide more details about the cell line

345: more details! Isolated from which animal/human, etc.

346: Is this the same cell line? Provide details

347: see 345

348: provide details about Vero-B4

342-349: Why did you used three different Vero cell lines? Explain in the text

350: how often do you performed this experiment? Add information

353: origin of blood?

351-354: how much percentages of what?

Table S9: is not necessary to provide this data in the manuscript, delete

360: Why do you choose this temperature? Explain! As far as average temperatures in Germany are concerned, the selected temperature seems to be too high. Why 85% humidity? This is also a very high humidity value, what´s the reason?

361: delete information about incubator, not necessary

368: how often do you performed this experiment? Add information

Fig 5: not necessary, as the workflow is also shown in Fig 4. Either delete Fig 4A or Fig 5.

392: there are too much details in this part, but important ones are missing: which qPCR-Kit/protocol did you use. It is not enough to cite a paper where it is mentioned.

402-403: Please mention the reason, citing is not enough

405 and 408-410: the sentences have the same meaning, delete one.

405-410: Add information how to calculate rates!

Reviewer #2: (No Response)

Reviewer #3: (No Response)

**********

**Part III – Minor Issues: Editorial and Data Presentation Modifications**

Please use this section for editorial suggestions as well as relatively minor modifications of existing data that would enhance clarity.

Reviewer #1: (No Response)

Reviewer #2: Results:

In table 1, it would be helpful to the reader to include a denotation of significance for the two Infection Rates.

Discussion:

Line 332 - Results do not necessarily indicate outcompeting only that there is inhibition of WNV infection.

Please include in the discussion the two following caveats - Well established mosquito colonies differ from field mosquito populations is a number of ways (genetics and gut microbiome) that may impact how the trends and results in this study translate.

- Sugar staving mosquitoes can impact blood feeding behavior that may complicate the results described in the study.

Methods:

Please briefly describe how you determined which of the two viruses was infectious in the presence of both WNV and USUV RNA (Ln 186). It can be found in the key at the bottom of S7 and S8 tables, but should be made clear in the manuscript.

Reviewer #3: 1. Title: leave out ‘viral wingmen’. In my opinion, it has no logical link with the topic of the study. Without this short ‘popular’ part, the title covers the content of the paper nicely.

2. Abstract, line 20: replace ‘medicine’ with ‘health’.

3. The abstract mentions that ‘…mosquitoes with established co-infections after simultaneous and sequential co-exposure exhibited higher WNV viral loads.’. While this is indeed apparent for simultaneous exposure (Figure 2E), I do not see this indicated for the sequential exposure data (Figure 4E). However, the text in lines 226-227 mention that ‘mosquitoes with established co-infection exhibited higher WNV load than those with single WNV infections, p = 0.027). How is this difference visible in Figure 4E? I had some trouble in understanding what was compared with what.

4. Line 30: ‘then’ should read ‘than’

5. Line 64/65: remove ‘the’ before the virus names

6. Results section: in many instances, just the p-values are reported without providing further details on the outcomes of the actual statistics. These details should be included throughout the results.

7. L.156: if it is not significantly different, you cannot claim that ‘WNV was transmitted more frequently’. This could be co-incidence as confirmed by your statistical test.

8. Figure 3: make the difference between panels B and C clear in the Y-axis labels. Both now mention USUV positivity rate, but B is for bodies and C is for saliva.

9. L. 210: here the authors mention that susceptibility to USUV was significantly reduced, but without providing any further statistical details on the outcome of the test (and the type of test performed).

10. L. 341: mention passage number of the viral strains used here.

11. L. 341: why did the authors chose to use the molestus biotype? In literature, it is not really considered an enzootic vector of USUV or WNV and there is evidence that its vector competence is different (lower) for these viruses in comparison with the pipiens biotype. A critical discussion on this is missing.

12. L 343: add ‘The’ before ‘rearing procedure’

**********

PLOS authors have the option to publish the peer review history of their article (what does this mean?). If published, this will include your full peer review and any attached files.

If you choose “no”, your identity will remain anonymous but your review may still be made public.

**Do you want your identity to be public for this peer review?**  For information about this choice, including consent withdrawal, please see our Privacy Policy.

Reviewer #1: No

Reviewer #2: No

Reviewer #3: No

[NOTE: If reviewer comments were submitted as an attachment file, they will be attached to this email and accessible via the submission site. Please log into your account, locate the manuscript record, and check for the action link "View Attachments". If this link does not appear, there are no attachment files.]

**Figure resubmission:**

While revising your submission, we strongly recommend that you use PLOS’s NAAS tool (https://ngplosjournals.pagemajik.ai/artanalysis) to test your figure files. NAAS can convert your figure files to the TIFF file type and meet basic requirements (such as print size, resolution), or provide you with a report on issues that do not meet our requirements and that NAAS cannot fix.

After uploading your figures to PLOS’s NAAS tool - https://ngplosjournals.pagemajik.ai/artanalysis, NAAS will process the files provided and display the results in the "Uploaded Files" section of the page as the processing is complete. If the uploaded figures meet our requirements (or NAAS is able to fix the files to meet our requirements), the figure will be marked as "fixed" above. If NAAS is unable to fix the files, a red "failed" label will appear above. When NAAS has confirmed that the figure files meet our requirements, please download the file via the download option, and include these NAAS processed figure files when submitting your revised manuscript.

**Reproducibility:**

To enhance the reproducibility of your results, we recommend that authors of applicable studies deposit laboratory protocols in protocols.io, where a protocol can be assigned its own identifier (DOI) such that it can be cited independently in the future. Additionally, PLOS ONE offers an option to publish peer-reviewed clinical study protocols. Read more information on sharing protocols at https://plos.org/protocols?utm_medium=editorial-email&utm_source=authorletters&utm_campaign=protocols

# Author comment: Author response to Decision Letter 1

**DOI:** 10.1371/journal.ppat.1014601.r002

23 Jul 2026

Attachment Submitted filename: Response to Reviewers.docx

# Peer review: Decision Letter 1

**DOI:** 10.1371/journal.ppat.1014601.r003

**Authors:** Matthias Schnell, Alan Goodman

25 Aug 2026

PPATHOGENS-D-26-00495R1

Usutu virus promotes West Nile virus replication in Culex pipiens biotype molestus during simultaneous and sequential co-infections

PLOS Pathogens

Dear Dr. Schäfer,

Thank you for submitting your manuscript to PLOS Pathogens. After careful consideration, we feel that it has merit but does not fully meet PLOS Pathogens's publication criteria as it currently stands. Therefore, we invite you to submit a revised version of the manuscript that addresses the points raised during the review process.

Please submit your revised manuscript by Oct 24 2026 11:59PM. If you will need more time than this to complete your revisions, please reply to this message or contact the journal office at plospathogens@plos.org. When you're ready to submit your revision, log on to https://www.editorialmanager.com/ppathogens/ and select the 'Submissions Needing Revision' folder to locate your manuscript file.

Please include the following items when submitting your revised manuscript:

* A letter that responds to each point raised by the editor and reviewer(s). You should upload this letter as a separate file labeled 'Response to Reviewers'. This file does not need to include responses to any formatting updates and technical items listed in the 'Journal Requirements' section below.

* A marked-up copy of your manuscript that highlights changes made to the original version. You should upload this as a separate file labeled 'Revised Manuscript with Track Changes'.

* An unmarked version of your revised paper without tracked changes. You should upload this as a separate file labeled 'Manuscript'.

If you would like to make changes to your financial disclosure, competing interests statement, or data availability statement, please make these updates within the submission form at the time of resubmission. Guidelines for resubmitting your figure files are available below the reviewer comments at the end of this letter.

As the corresponding author, your ORCID iD is verified in the submission system and will appear in the published article. PLOS supports the use of ORCID, and we encourage all coauthors to register for an ORCID iD and use it as well. Please encourage your coauthors to verify their ORCID iD within the submission system before final acceptance, as unverified ORCID iDs will not appear in the published article. Only the individual author can complete the verification step; PLOS staff cannot verify ORCID iDs on behalf of authors.

We look forward to receiving your revised manuscript.

Kind regards,

Alan G. Goodman

Academic Editor

PLOS Pathogens

Matthias Schnell

Section Editor

PLOS Pathogens

Sumita Bhaduri-McIntosh

Editor-in-Chief

PLOS Pathogens

orcid.org/0000-0003-2946-9497

Dominique Soldati-Favre

Editor-in-Chief

PLOS Pathogens

orcid.org/0000-0003-4156-2109

**Additional Editor Comments (if provided):**

Thank you for submitting your revised manuscript. Please address the remaining minor concerns from the reviewers.

**Journal Requirements:**

If the reviewer comments include a recommendation to cite specific previously published works, please review and evaluate these publications to determine whether they are relevant and should be cited. There is no requirement to cite these works unless the editor has indicated otherwise.

[Note: HTML markup is below. Please do not edit.]

**Reviewers' Comments:**

Reviewer's Responses to Questions

**Part I - Summary**

Please use this section to discuss strengths/weaknesses of study, novelty/significance, general execution and scholarship.

Reviewer #2: This paper fills in knowledge gaps surrounding Usutu virus and West Nile virus within vector biology. The previous works the authors are building their work around have found vector species and virus strain specific interactions, and the novel aspect of this manuscript lies in the inconsistencies that occur using a different lineage of virus and biotype of Culex pipiens. While the field relevance of these findings are not explicit, due to the limited data surrounding co-infections, the spotlight this work shines on the specificity of these interactions is important for assumptions made about other virus/mosquito interactions that may change with origin.

Reviewer #3: This is a revision of a manuscript that I reviewed earlier. Substantial changes have been made to the different sections of the manuscript in response to the three reviewers. In general, results and conclusions have been more precisely and more carefully formulated now. Although the novelty of the presented work remains limited, the work provides evidence of the effects of co-exposure on WNV and USUV dynamics inside the mosquito. I have no further suggestions for modifications.

**********

**Part II – Major Issues: Key Experiments Required for Acceptance**

Please use this section to detail the key new experiments or modifications of existing experiments that should be absolutely required to validate study conclusions.

Generally, there should be no more than 3 such required experiments or major modifications for a "Major Revision" recommendation. If more than 3 experiments are necessary to validate the study conclusions, then you are encouraged to recommend "Reject".

Reviewer #2: (No Response)

Reviewer #3: -

**********

**Part III – Minor Issues: Editorial and Data Presentation Modifications**

Please use this section for editorial suggestions as well as relatively minor modifications of existing data that would enhance clarity.

Reviewer #2: Lines 73-77: In the section, as written there appears to be two human blood donors with co-infections of WNV and USUV. However, when digging into the citation, both lead to the same case. Please rewrite to clarify the number of recorded human co-infections.

Lines 79- 82: These sentences deliver the same information. They are be combined to increase reader clarity.

Reviewer #3: -

**********

PLOS authors have the option to publish the peer review history of their article (what does this mean?). If published, this will include your full peer review and any attached files.

If you choose “no”, your identity will remain anonymous but your review may still be made public.

**Do you want your identity to be public for this peer review?**  For information about this choice, including consent withdrawal, please see our Privacy Policy.

Reviewer #2: No

Reviewer #3: No

[NOTE: If reviewer comments were submitted as an attachment file, they will be attached to this email and accessible via the submission site. Please log into your account, locate the manuscript record, and check for the action link "View Attachments". If this link does not appear, there are no attachment files.]

**Figure resubmission:**

While revising your submission, we strongly recommend that you use PLOS’s NAAS tool (https://ngplosjournals.pagemajik.ai/artanalysis) to test your figure files. NAAS can convert your figure files to the TIFF file type and meet basic requirements (such as print size, resolution), or provide you with a report on issues that do not meet our requirements and that NAAS cannot fix.

After uploading your figures to PLOS’s NAAS tool - https://ngplosjournals.pagemajik.ai/artanalysis, NAAS will process the files provided and display the results in the "Uploaded Files" section of the page as the processing is complete. If the uploaded figures meet our requirements (or NAAS is able to fix the files to meet our requirements), the figure will be marked as "fixed" above. If NAAS is unable to fix the files, a red "failed" label will appear above. When NAAS has confirmed that the figure files meet our requirements, please download the file via the download option, and include these NAAS processed figure files when submitting your revised manuscript.

**Reproducibility:**

To enhance the reproducibility of your results, we recommend that authors of applicable studies deposit laboratory protocols in protocols.io, where a protocol can be assigned its own identifier (DOI) such that it can be cited independently in the future. Additionally, PLOS ONE offers an option to publish peer-reviewed clinical study protocols. Read more information on sharing protocols at https://plos.org/protocols?utm_medium=editorial-email&utm_source=authorletters&utm_campaign=protocols

# Author comment: Author response to Decision Letter 2

**DOI:** 10.1371/journal.ppat.1014601.r004

31 Aug 2026

Attachment Submitted filename: Letter of Response.docx

# Editor report: Decision Letter 2

**DOI:** 10.1371/journal.ppat.1014601.r005

**Authors:** Matthias Schnell, Alan Goodman

4 Sep 2026

Dear Schäfer,

We are pleased to inform that your manuscript, "Usutu virus promotes West Nile virus replication in Culex pipiens biotype molestus during simultaneous and sequential co-infections", has been editorially accepted for publication at PLOS Pathogens.

Before your manuscript can be formally accepted and sent to production, you will need to complete our formatting changes, which you will receive by email within a week. Please note that your manuscript will not be scheduled for publication until you have made the required changes.

IMPORTANT NOTES

(1) Copyediting and Proofreading: The corresponding author will receive a typeset proof for review, to ensure errors have not been introduced during production. Please review the PDF proof of your manuscript carefully, as this is the last chance to correct any errors. Please note that major changes, or those which affect the scientific understanding of the work, will likely cause delays to the publication date of your manuscript.

(2) Appropriate Figure Files: Please remove all name and figure # text from your figure files. Please also take this time to check that your figures are of high resolution, which will improve the readbility of your figures and help expedite your manuscript's publication. Please note that figures must have been originally created at 300dpi or higher. Do not manually increase the resolution of your files. For instructions on how to properly obtain high quality images, please review our Figure Guidelines, with examples at: http://journals.plos.org/plospathogens/s/figures.

We encourage you to use PACE (http://pace.apexcovantage.com/), a figure-checking tool which helps authors ensure all figures meet PLOS requirements so that figures can publish in the highest quality. PACE is a digital diagnostic and conversion tool for figure files. It will provide information about any failed check(s) and, if able, will automatically convert the figure into an acceptable file that passes quality checks.

To use PACE, you must first register as a user. Then, login and navigate to the UPLOAD tab, where you will find detailed instructions on how to use the tool.

(3) Striking Image: Please upload a striking still image to accompany your article if one is available (you can include a new image or an existing one from within your manuscript). Should your paper be accepted, this image will be considered for our monthly issue image and may also appear on our website to feature your article. Please upload this as a separate file, selecting "striking image" as the file type upon upload. Please also include a separate "Other" file with a caption, including credits and any potential copyright information. Please do not include the caption in the main article file. If your image is from someone other than yourself, please ensure that the artist has read and agreed to the terms and conditions of the Creative Commons Attribution License at http://journals.plos.org/plospathogens/s/content-license. Please note that PLOS cannot publish copyrighted images.

(4) Press Release or Related Media: If you or your institution will be preparing press materials for this manuscript, please inform our press team in advance at plospathogens@plos.org as soon as possible. We ask that you contact us within one week to plan ahead of our fast Production schedule. If you need to know your paper's publication date for related media purposes, you must coordinate with our press team, and your manuscript will remain under a strict press embargo until the publication date and time.

(5) Update your Profile Information: Now that your manuscript has been provisionally accepted, please log into Editorial Manager and update your profile, if needed. Go to http://www.editorialmanager.com/ppathogens, log in, and click on the "Update My Information" link at the top of the page. Please update your user information to ensure an efficient production and billing process.

(6) LaTeX users only: Our staff will ask you to upload a TEX file in addition to the PDF before the paper can be sent to typesetting, so please carefully review our Latex Guidelines http://journals.plos.org/plospathogens/s/latex in the meantime.

Thank you again for supporting Open Access publishing; we are looking forward to publishing your work in PLOS Pathogens.

Best regards,

Alan G. Goodman

Academic Editor

PLOS Pathogens

Matthias Schnell

Section Editor

PLOS Pathogens

Dominique Soldati-Favre

Editor-in-Chief

PLOS Pathogens

***********************************************************

# Editor report: Acceptance letter

**DOI:** 10.1371/journal.ppat.1014601.r006

**Authors:** Matthias Schnell, Alan Goodman

Dear Schäfer,

We are delighted to inform you that your manuscript, "Usutu virus promotes West Nile virus replication in Culex pipiens biotype molestus during simultaneous and sequential co-infections," has been formally accepted for publication in PLOS Pathogens.

We have now passed your article onto the PLOS Production Department who will complete the rest of the pre-publication process. All authors will receive a confirmation email upon publication.

Thank you again for supporting open-access publishing; we are looking forward to publishing your work in PLOS Pathogens.

Best regards,

Dominique Soldati-Favre

Editor-in-Chief

PLOS Pathogens
